# Reoperation for failed shoulder reconstruction following brachial plexus birth injury

**DOI:** 10.1186/1749-7221-8-7

**Published:** 2013-07-25

**Authors:** Andrew E Price, Marc Fajardo, John AI Grossman

**Affiliations:** 1 Department of Orthopaedic Surgery, NYU Hospital for Joint Diseases, New York, USA; 2 Brachial Plexus and Peripheral Nerve Programs, Miami Children’s Hospital, Miami, FL, USA

**Keywords:** Brachial plexus birth palsy, Reoperation, Revision surgery, Reconstruction

## Abstract

**Background:**

Various approaches have been developed to treat the progressive shoulder deformity in patients with brachial plexus birth palsy. Reconstructive surgery for this condition consists of complex procedures with a risk for failure.

**Case presentations:**

This is a retrospective case review of the outcome in eight cases referred to us for reoperation for failed shoulder reconstructions. In each case, we describe the initial attempt(s) at surgical correction, the underlying causes of failure, and the procedures performed to rectify the problem. Results were assessed using pre- and post-operative Mallet shoulder scores. All eight patients realized improvement in shoulder function from reoperation.

**Conclusions:**

This case review identifies several aspects of reconstructive shoulder surgery for brachial plexus birth injury that may cause failure of the index procedure(s) and outlines critical steps in the evaluation and execution of shoulder reconstruction.

## Level of evidence: IV

The most common musculoskeletal deformity following brachial plexus birth injury is medial rotation contracture of the shoulder, which is often coupled with a limitation of shoulder elevation. In these patients, the lateral rotators of the shoulder are relatively more affected by the injury than the medial rotators. Additionally, the subscapularis is weakened by the injury and becomes secondarily contracted due to the relative sparing of the other powerful internal rotators [1]. In patients who have a longstanding contracture, bony incongruence and posterior subluxation/dislocation will develop in the glenohumeral joint [2,3]. When such glenohumeral deformity is present, a lateral rotation osteotomy of the humerus is the only option to gain a more functional arc of motion and improve limb condition [4]. If the patient is seen before this permanent bony deformity occurs, release of the contracture and tendon transfers will improve shoulder function and appearance. The window of opportunity for the approach using the release and transfers depends on the presence of a congruent glenohumeral joint [5]. The latest time for release and transfer has not been established. If left untreated the glenohumeral joint deformity will progress over time, correlating to the magnitude of the medial rotation contracture [3].

Various approaches have been developed to treat the progressive shoulder deformity in these patients. Surgical release of the medial rotation contracture was first described by Fairbank [6] in 1913 and Sever [7] in 1918. They reported direct release of the subscapularis and pectoralis at their insertions on the humerus [6,7]. Many surgeons continue to perform the release anteriorly at the insertion. Others have elected to accomplish the release at the origin of the subscapularis on the scapula (subscapularis slide), which was first described by Carlioz and Brahini [8]. Tendon transfers are often performed in addition to the release of the contracture in order to prevent recurrence of the medial rotation contracture due to the inherent muscle imbalance, depending on the age of the patient and/or time since a primary plexus repair. L’Episcopo combined the anterior release with transfer of the teres major posteriorly to act as a lateral rotator [9]. He later transferred the latissimus dorsi together with the teres major [10]. Hoffer et al. [11] reattached this conjoined tendon high up on the posterior humeral head into the infraspinatus tendon. With concomitant release of the pectoralis major, function was restored and proper shoulder joint position maintained. Several others have reported excellent results with increases in lateral rotation and abduction [12,13]. Modifications in the original technique have been made, primarily in how and where the transferred tendon is reattached [13].

Despite their apparent technical simplicity, reconstructive shoulder surgeries due to brachial plexus birth palsy are in fact complex procedures with a significant risk of failure. We report eight consecutive cases in which reoperation was required for failed shoulder reconstructions. In each case we describe initial procedure based on a review of available operative records, an analysis of the underlying causes of failure, and procedures performed to rectify the problem (Table 1). Pre-operative and post-operative shoulder elevation and Mallet scores were used to evaluate our results (Table 2). To our knowledge this is the only reported series of patients undergoing reoperation in this clinical scenario.

**Table 1:** Operative history and technical errors

**Patient**	**Previous surgery**	**Operative correction**	**Technical error**
1	Brachial plexus exploration, sural nerve grafts C3, C4, C5 to C7, SAN transfer to C7; teres major transfer to teres minor, partial acromion excision, external neurolysis axillary, radial, ulnar, musculocutaneous, thoracodorsal, long thoracic nerves; proximal humeral osteotomy, pectoralis major lengthening	Latissimus dorsi transfer into infraspinatus	Injured neurovascular Pedicle to teres major
2	Modified quad, including subscapularis release, teres major to teres minor transfer, neurolysis axillary nerve, pectoralis release	Latissimus dorsi transfer, transfer of sternal head of pectoralis major to lesser tuberosity	Loss of subscapularis power, devascularization of transferred teres major
3	L’Episcopo procedure	External rotation osteotomy	Failed muscle transfer
4	Exploration and nerve grafting; modified quad x 2	Humeral external rotation osteotomy	Failed muscle transfer
5	External rotational osteotomy of the humerus	Internal humeral osteotomy	Excessive external rotation of original osteotomy
6	(1) External neurolysis; (2) neurolysis of axillary nerve, transfer of teres major, release of subscapularis, pectoralis major and minor	External rotation osteotomy pectoralis major release	Failed muscles transfer
7	Exploration, neurolysis, nerve grafting; modified quad, including teres major transfer to teres minor, release of pectoralis minor, biceps short head, pectoralis major lengthening, neurolysis axillary nerve	Subscapularis slide, intramuscular lengthening pectoralis major, latissimus dorsi transfer, repair of teres major	Inadequate placement of transferred muscle
8	Release of subscapularis, proximal triceps, pectoralis, teres major, shoulder capsule neurolysis axillary nerve	Transfer of clavicular head of pectoralis to greater tuberosity	Complete disruption and loss of subscapularis power

**Table 2:** Pre- and postoperative data

**Case**	**Pre-op elevation**	**Post-op elevation**	**Pre-mallet**	**Post-mallet**
1	15	30	13	14
2	45	90	11	17
3	100	150	13	16
4	165	165	14	18
5	80	80	11	14
6	90	140	12	18
7	45	90	15	18
8	120	170	13	18

### Case 1

AW was first seen at age 6 years and 5 months with severe sequelae from a right global plexus palsy. At 10 months of age she had undergone exploration with neurolysis of the supra- and infraclavicular brachial plexuses, a spinal accessory nerve–to-suprascapular nerve transfer, and a C3/C4/C5 motor branch nerve transfer to the C7 root using a sural nerve interposition graft. At the age of 4 years and 4 months, she was seen by a different physician at another medical center where she underwent a teres major muscle transfer to the teres minor, posterior shoulder capsulorrhaphy, and shaving/excision of an acromial bone spur. When she was 5 years and 8 months, she underwent rotational osteotomy of the humerus by yet another physician. When she was 6 years and 5 months, her mother brought her to our center because she was still having considerable problems with extremity function.

**Figure 1:**
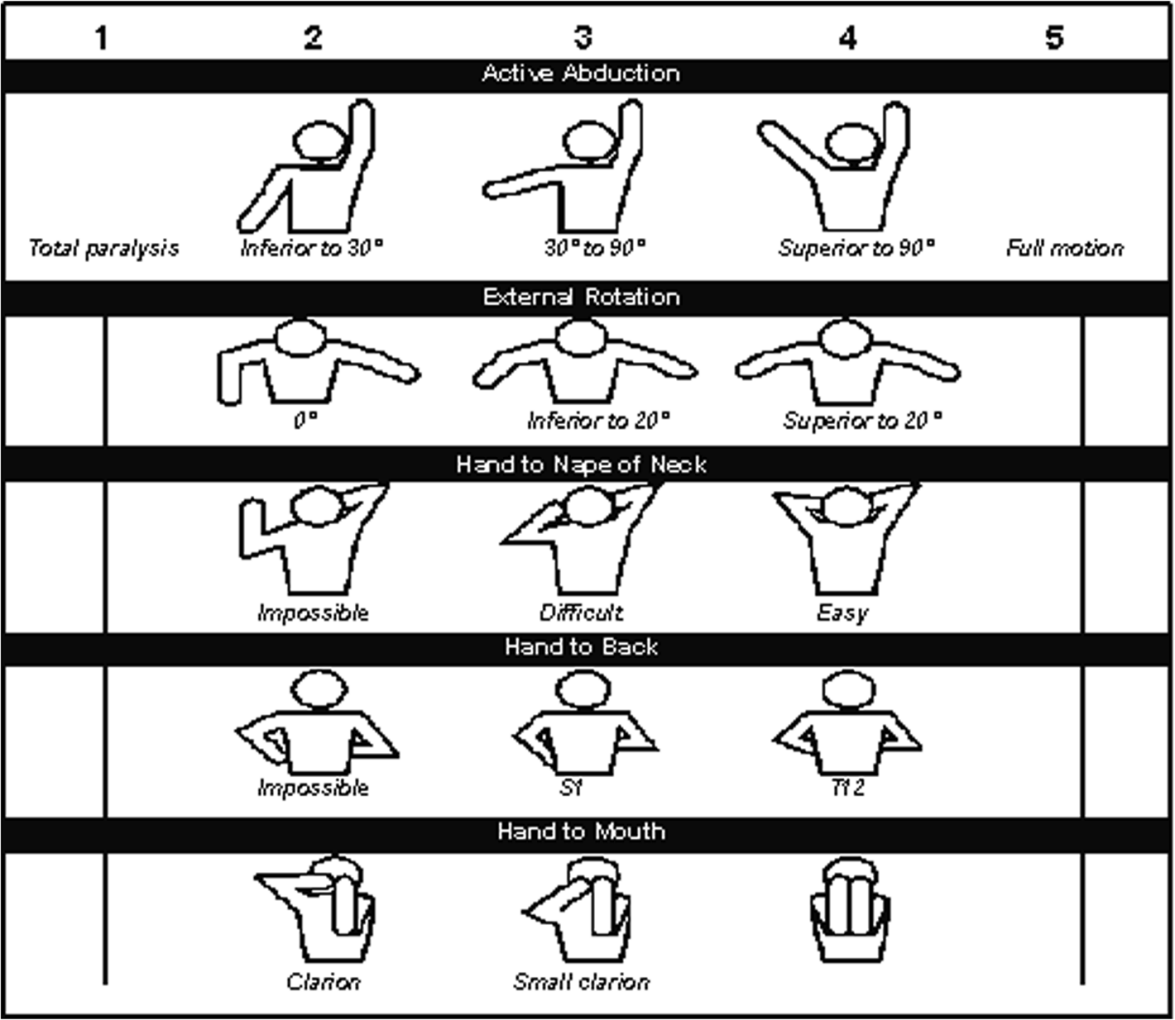
**Mallet classification of shoulder following obstetrical brachial plexus injury.** Total score from all columns: 0–4 indicates minimal function (grade 0); 5–9, poor (grade 1); 10–13, fair (grade 2); 14–17, satisfactory (grade 3); 18–22, good (grade 4); and 22–25, excellent (grade 5). (Adapted from Grossman JAI, Ramos LE, Sumway S, Alfonso I. Management strategies for children with obstetrical brachial plexus injuries. Int Pediatr 1997;12:82–86.)

Physical examination of the right upper extremity demonstrated transverse scars overlying the acromion and the axilla, a longitudinal scar over the anteromedial arm, and a visual deformity of the upper arm. She had active shoulder elevation to 80 degrees with a positive clarion or trumpet sign. With the arm adducted, she had no active lateral rotation power; her passive shoulder motion in adduction was from 60 degrees of lateral rotation to 90 degrees of medial rotation. Her Mallet score was 11 (Figure 1) [14,15].

At age 6 years and 8 months, re-exploration revealed that the teres major was severely atrophic and had been detached from its neurovascular pedicle leaving only a few healthy muscle fibers not included in the transfer. The tendinous portion of the muscle was mainly scar tissue that ended at the posterior margin of the deltoid. We transferred the untouched latissimus dorsi muscle high up on the infraspinatus. At latest follow-up at 1 year post-surgery she demonstrated 100 degrees of shoulder elevation with no posturing of the extremity at rest. She had a negative clarion sign. Her postoperative Mallet score was 17. Her father reported increased subjective use of the extremity as compared with her preoperative function.

### Case 2

GC was first seen at age 10 years and 8 months with a right brachial plexus birth injury. Four and a half years prior she had undergone a release of medial rotation contracture of her right shoulder by a subscapularis tenotomy through an axillary approach, and transfer of the teres major muscle under the long head of the triceps into the teres minor. She had active shoulder elevation to 45 degrees with a negative clarion sign. She was unable to actively medially rotate her shoulder and could not reach her abdomen with her right hand. She had only 45 degrees of active lateral rotation and an elbow flexion contracture of 25 degrees, but demonstrated good recovery of her biceps, triceps, forearm, and hand. Her Mallet score was 16. She subsequently underwent exploration of her shoulder through her previous axillary incision. We noted that the teres major was badly atrophic, containing extensive fatty degeneration with a portion of the tendon extending under the long head of the triceps but ending abruptly. The neurovascular pedicle to the atrophic teres major could not be identified. The intact latissimus dorsi tendon was freed of all adhesions, detached from the anterior humerus, and passed over the long head of the triceps under the posterior edge of the deltoid and transferred into the infraspinatus tendon. We then made a seven-centimeter incision over the deltopectoral groove and dissected the sternal head from the clavicular head of the pectoralis major and detached the former from its insertion into the humerus. The sternal head was then passed under the conjoined tendon, inferior to the coracoids, and sewn into the lesser tuberosity. One year later she was able to bring her hand to her waist and button her pants. Her shoulder elevation had increased to 90 degrees. Her Mallet score increased to 18.

### Case 3

ND was a 10-and-a-half-year-old girl with a history of left Erb’s palsy. Slightly before her fourth birthday, she underwent a L’Episcopo procedure, was casted for 8 weeks, and underwent physical therapy. On examination, her left upper extremity demonstrated a medial rotation contracture with shoulder elevation to 170 degrees, a positive clarion sign, and inability to supinate the forearm. Her shoulder lateral rotation with the arm adducted was 10 degrees and she could medially rotate to 120 degrees. Her Mallet score was 15. Her previous transfer was not explored, but we performed a lateral rotation osteotomy. At 1-year post-surgery, she has 170 degrees of shoulder elevation, no clarion sign, and improved posture of her arm. Her Mallet score improved to 19.

### Case 4

CA presented at age 6 years and 4 months with sequalae of a right global brachial plexus birth palsy. As an infant, he underwent exploration, nerve grafting (exact procedure unknown), and two subsequent secondary surgeries including a teres major transfer, subscapularis release, and axillary nerve decompression, all at other institutions. He presented with a severely hypoplastic right upper extremity postured in medial rotation, elbow flexion, and hyperpronation. He exhibited a severe clarion sign, was unable to get his hand to his face, and had active shoulder elevation to 80 degrees. His passive lateral rotation of the shoulder was 0 degrees, and he could medially rotate the shoulder to 95 degrees. He had a 45-degree elbow flexion contracture with an anteriorly dislocated radial head. Computed tomography of the shoulder revealed a hypoplastic, dislocated glenohumeral joint. His Mallet score was 12. We performed a lateral rotation osteotomy of the humerus. At 2 years follow-up, he was using his right hand to play video games and operate a computer. His Mallet score was 17.

### Case 5

SG was a 7-year-old boy with sequalae of a global plexus palsy. At age 6 he had undergone a lateral rotation osteotomy of the humerus. He presented with limitations of shoulder medial rotation and was unable to bring his hand to his waist. Passively, he had full lateral rotation of the shoulder. He had 30 degrees of active shoulder elevation, which was thought to be mostly scapulothoracic. His preoperative Mallet score was 13. He subsequently had a revision of the rotational osteotomy to gain medial rotation. At 7 years follow-up, the patient had forward elevation to 30 degrees and was able to bring his hand to his waist. He had active medial rotation to 75 degrees and lateral rotation to 70 degrees. His Mallet score was 14.

### Case 6

MS was a 10-year-old boy with a global left brachial plexus palsy. At 16 months of age, he underwent neurolysis of the brachial plexus. At age 4 years, he underwent release of the subscapularis and pectoralis muscles, axillary nerve neurolysis in the quadrilateral space, and transfer of the latissimus and teres major muscles. He presented to our center at age 10 with left shoulder internal rotation, active shoulder elevation to 100 degrees, and a positive clarion sign. His preoperative Mallet score was 13. Magnetic resonance imaging demonstrated a dysplastic, posteriorly dislocated glenohumeral joint. He underwent a lateral rotational osteotomy of the humerus and release of the pectoralis major muscle. His 1-year postoperative Mallet score was 16 and he demonstrated forward elevation to 150 degrees.

### Case 7

JH was a 12-year-old girl who presented to our center with left Erb’s Palsy. At 8 months of age she had undergone left brachial plexus surgical exploration with neurolysis and sural nerve grafting from the C5 nerve root. At age 7, she had undergone a teres major to teres minor transfer, release of the pectoralis minor, release of the short head of the biceps, pectoralis major lengthening, and axillary nerve neurolysis in the quadrilateral space. She presented with a positive clarion sign with her arm postured in medial rotation. Her active shoulder elevation was 90 degrees. Her passive lateral rotation with her arm adducted was to negative 10 degrees. Her preoperative Mallet score was 12. Exploration of her axilla revealed that the transferred teres major was scarred to the subcutaneous tissue and the inferior edge of the teres minor muscle. A left subscapularis slide, intramuscular lengthening of the pectoralis major, transfer of the latissimus dorsi to the infraspinatus, and repair of the teres major were performed. At 1-year postsurgery, she was able to elevate the shoulder to 140 degrees; her Mallet score was 18.

### Case 8

**Figure 2:**
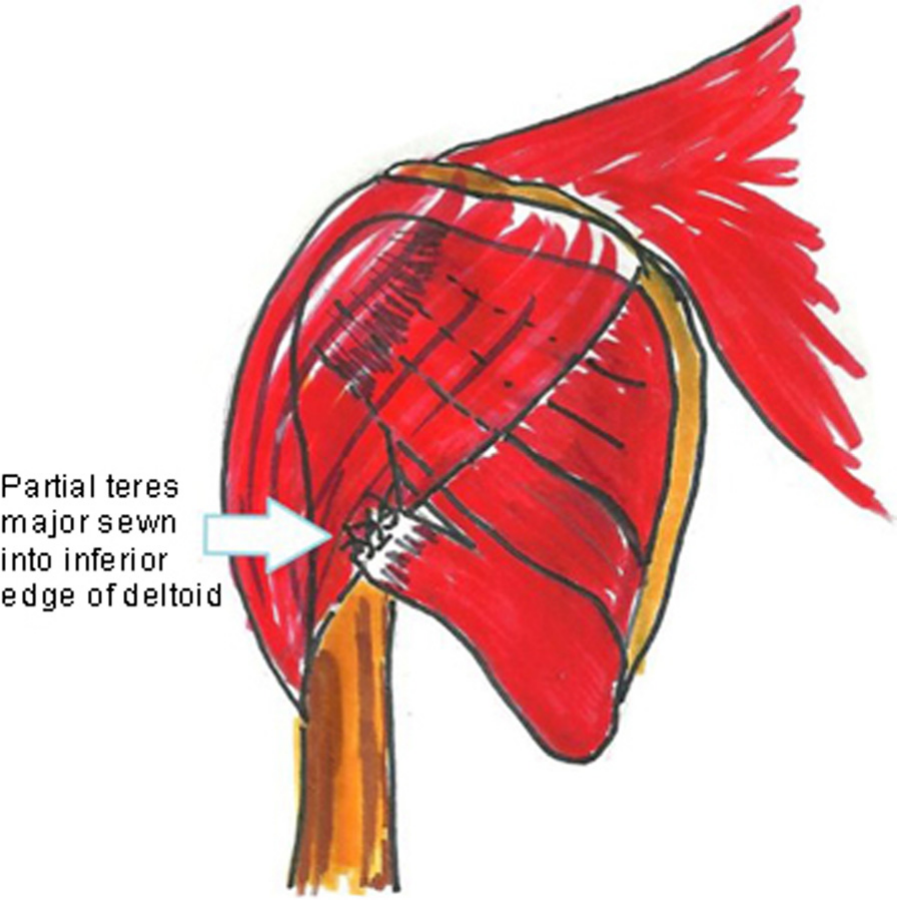
Example of faulty harvest and insertion of transferred tendon.

RS was a 4-year-old boy who presented with right Erb’s palsy. At age 6 months he had undergone brachial plexus exploration with nerve grafting. The details of the operation were not available for review. At 18 months of age, he underwent open anterior subscapularis release, transfer of the teres major, axillary nerve neurolysis, and pectoralis major release. At age 6, he had shoulder elevation to 120 degrees with a severe clarion sign. His passive lateral rotation of the shoulder in adduction was to 0 degrees; his Mallet score was 13. At exploration, the teres major was split in half and attached to the posterior edge of the deltoid (Figure 2). We performed a subscapularis slide and repaired the teres major to the rest of the muscle which had remained in its anatomic site. The latissimus dorsi was detached from the humerus and transferred into the infraspinatus tendon. At 18 months postsurgery, he had 170 degrees of active shoulder elevation and a negative clarion sign. His postoperative Mallet score was 18.

## Discussion

Over the last 25 years there has been increased interest in surgical reconstruction for shoulder sequalae of brachial plexus birth injury. Our analysis of eight reoperated cases identified several problem areas, which may help guide planning and execution of these cases. They include preoperative and intraoperative categories (Table 3). While the necessary criteria for release and transfer have not been established, we believe that success is less likely after the age of 4 years. In children younger than 4 years, we believe greater potential exists for the glenohumeral joint to remodel and, thus, we are inclined to release the contracture and do muscle transfers. In children over 4 years of age, preoperative evaluation of the congruity of the glenohumeral joint is essential to determine whether a release and transfer are possible. We obtain an MRI to assess the congruity and orientation of the glenohumeral joint. If MRI reveals a spherical, congruent, and reduced humeral head, a release of the internal rotation contracture with a muscle transfer is indicated. In a child older than 4 years with such a dysplastic joint, a rotational osteotomy is indicated. A humeral head that is aspherical and/or subluxed/dislocated with a dysplastic glenoid will not regain adequate external rotation, nor have the remodeling capability to correct the dysplasia. It is prone to “settle” back to its preoperative position. Humeral osteotomy is therefore indicated [16].

**Table 3:** Major preoperative and postoperative errors

**Preoperative errors**	**Posoperative errors**
1. Sphericity of humeral head	1. Incision placement
2. Dysplasia of glenoid	2. Incomplete release or restoration of lateral rotation
3. Severe glenoid retroversion	3. Excessive release of subscapularis
4. Strength of latissimus dorsi/teres major	4. Excessive lateral rotation in osteotomy
	5. Injury to muscular neurovascular pedicle
	6. Incorrect or poor insertion of transferred tendon
	7. Immobilization in excessive medial or lateral rotation

When contracture release and transfer are indicated, the internal rotation contracture is first released in a step-wise fashion to restore full passive external rotation of the shoulder in adduction. We prefer to first lengthen the subscapularis through a “slide” at its origin on the scapula. This avoids the potential for over-release that can occur with insertion through open or arthroscopic techniques (Case 2) [17]. If, after subscapularis slide, persistent tightness is present, the pectoralis major is lengthened intramuscularly. When subluxation or dislocation has been present for an extended period of time, we have encountered contracture of the coracohumeral ligament, the conjoin tendon, and an overgrown coracoid, which may prevent full reduction of the humeral head into the true glenoid. Thus, coracohumeral ligament release, partial release of the conjoint tendon, and excision of the tip of the coracoid may be necessary. We immobilize the shoulder in a modified shoulder spica with the arm adducted in 60 degrees of external rotation to ensure that we do not over-lengthen the subscapularis. We have never had to release the capsule or the subscapularis at its insertion.

**Figure 3:**
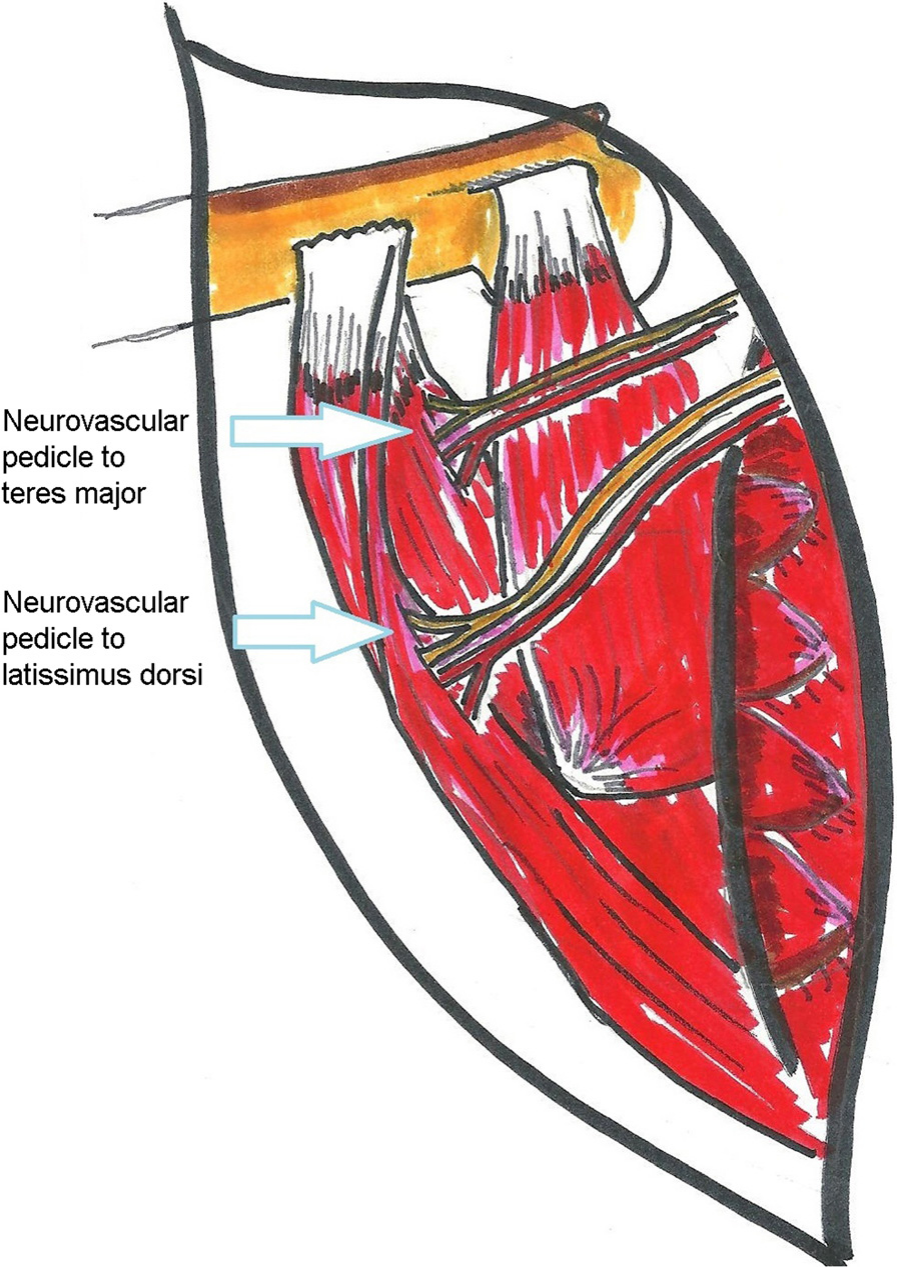
**Neurovascular pedicle to teres major must be identified and protected during transfer.** It is shorter and less mobile than the pedicle to latisimus dorsi.

A major soft tissue problem to be avoided is injury to the teres major neurovascular pedicle (Cases 1, 2, 5, and 7). When transferring the teres major (and/or the latissimus dorsi), it is important to identify and protect the neurovascular pedicles (Figure 3). Fortunately in these cases, the latissimus dorsi was still available for transfer. The transferred tendon should be brought over the long head of the triceps and sewn into the infraspinatus tendon to supplement external rotation and shoulder elevation [18]. We use at least 3 non-absorbable sutures to secure the transferred tendon. Immobilization in 60 degrees of external rotation and 30 degrees abduction is maintained for a period of 6 weeks, and the postoperative therapy program includes restoration of internal and external rotation, strengthening, and constraint-induced therapy.

If significant glenohumeral incongruity exists in a child older than 4 years, then a rotational osteotomy is performed. In patients with complete absence of external rotation power, we also perform a latissimus dorsi transfer. If rotational osteotomy is performed without any external rotation power, the patient will continue to exhibit a clarion sign and have difficulty raising the hand to the head.

Although under-correction may result in suboptimal functional improvements, excessive external rotation of the distal humerus may result in the inability to bring the ipsilateral hand to the waist, causing considerable limitations with personal hygiene and activities of daily living. Preoperatively, it is important to assess the arc of motion of the shoulder in adduction when planning the osteotomy. The rotation needs to be enough to remove the posturing of the shoulder and enable the patient to reach behind his/her head; however, one must still retain enough internal rotation to get to the umbilicus (Case 6). Usually, the osteotomy is rotated between 40 and 50 degrees. The osteotomy must be performed proximal to the deltoid tubercle to restore it to a more normal mechanical axis. Thus, the osteotomy is performed at a level between the pectoralis major insertion and the deltoid tubercle.

Whether a release or an osteotomy is performed, inability to actively bring the hand to the abdomen with or without an external rotation contracture is a significant functional problem. We have found recovery and reattachment of the subscapularis to be improbable in a non-acute setting. When the subscapularis has been “lost,” we have utilized a pectoralis major transfer if the patient has full passive internal rotation to their abdomen (Case 2). In patients where this deficit has been longstanding and passive rotation is lost, we perform an internal rotation osteotomy with enough rotation to “return” the hand to the umbilicus (Case 6).

Suboptimal placement of the transferred tendon on the shoulder is another complication. Correct placement of the transferred tendon is integral to establishing correct tension and subsequent function. Furthermore, we agree with Hoffer et al. [11] that transferring the muscle into the infraspinatus tendon utilizes the mechanical properties of the infraspinatus, with gains in both external rotation and elevation. We encountered seven patients with failure of the transferred tendon. The failure was found to be due to devascularization of the transferred muscle or suboptimal or loss of placement of the transferred tendon.

In summary, we report eight failed secondary reconstructions, detailing the pathology found at surgery and the steps taken to correct or improve prior failed procedures and outlining key points in the planning and execution of such procedures. All eight patients realized some improvement from our reconstructive attempts.

## Competing interest

The authors declare that they have no competing interests.

## Authors’ contribution

All authors contributed to the study design and preparation of the manuscript. All authors read and approved the final manuscript.

## Financial disclosure

The authors have no financial disclosures.

## References

[B1] Poyhia TH Nietosvaara YA Remes VM Kirjavainen MO Peltonen JI Lamminen AE MRI of rotator cuff muscle atrophy in relation to glenohumeral joint incongruence in brachial plexus birth injury Pediatr Radiol 2005 8 4 402 409 10.1007/s00247-004-1377-3 15635469

[B2] Hogendoorn S van Overvest KL Watt I Duijsens AH Nelissen RG Structural changes in muscle and glenohumeral joint deformity in neonatal brachial plexus palsy J Bone Joint Surg Am 2010 8 4 935 942 10.2106/JBJS.I.00193 20360518

[B3] Pearl ML Edgerton BW Glenoid deformity secondary to brachial plexus birth palsy J Bone Joint Surg Am 1998 8 5 659 667 9611026 10.2106/00004623-199805000-00006

[B4] Hale HB Bae DS Waters PM Current concepts in the management of brachial plexus birth palsy J Hand Surg Am 2010 8 2 322 331 10.1016/j.jhsa.2009.11.026 20141905

[B5] Price A Tidwell M Grossman JA Improving shoulder and elbow function in children with Erb's palsy Semin Pediatr Neurol 2000 8 1 44 51 10.1016/S1071-9091(00)80009-1 10749513

[B6] Fairbank H A lecture on birth palsy: subluxation of the shoulder-joint in infants and young children Lancet 1913 8 4679 1217 1223 10.1016/S0140-6736(00)52017-0

[B7] Sever J The results of a new operation for obstetrical paralysis Am J Orthop Surg 1918 8 248 257

[B8] Carlioz H Brahimi L Place of internal disinsertion of the subscapularis muscle in the treatment of obstetric paralysis of the upper limb in children Ann Chir Infant 1971 8 159 167 5562757

[B9] L’Episcopo J Tendon transplantation in obstetrical paralysis Am J Surg 1934 8 122 10.1016/S0002-9610(34)90143-4

[B10] L’Episcopo J Restoration of muscle balance in the treatment of obstetrical paralysis NY State J Med 1939 8 357

[B11] Hoffer MM Wickenden R Roper B Brachial plexus birth palsies Results of tendon transfers to the rotator cuff. J Bone Joint Surg Am 1978 8 5 691 695 681392

[B12] Phipps GJ Hoffer MM Latissimus dorsi and teres major transfer to rotator cuff for Erb's palsy J Shoulder Elbow Surg 1995 8 2 124 129 10.1016/S1058-2746(05)80066-7 7600163

[B13] Safoury Y Muscle transfer for shoulder reconstruction in obstetrical brachial plexus lesions Handchir Mikrochir Plast Chir 2005 8 5 332 336 10.1055/s-2005-872818 16287018

[B14] Mallet J Primaut’e du traitement de l”epaule—m’ethode d’expression des r’esultats. [Obstetrical paralysis of the brachial plexus. II. Therapeutics. Treatment of sequelae. Priority for the treatment of the shoulder. Method for the expression of results] Rev Chir Orthop Reparatrice Appar Mot 1972 8 166 168 4263979

[B15] Grossman JA DiTaranto P Yaylali I Alfonso I Ramos LE Price AE Shoulder function following late neurolysis and bypass grafting for upper brachial plexus birth injuries J Hand Surg Br 2004 8 4 356 358 10.1016/J.JHSB.2004.03.008 15234499

[B16] Price AE Grossman JA A management approach for secondary shoulder and forearm deformities following obstetrical brachial plexus injury Hand Clin 1995 8 4 607 617 8567742

[B17] Kirjavainen M Remes V Peltonen J Kinnunen P Pöyhiä T Telaranta T Alanen M Helenius I Nietosvaara Y Long-term results of surgery for brachial plexus birth palsy J Bone Joint Surg Am 2007 8 1 18 26 10.2106/JBJS.E.00430 17200305

[B18] Pearl ML Edgerton BW Kazimiroff PA Burchette RJ Wong K Arthroscopic release and latissimus dorsi transfer for shoulder internal rotation contractures and glenohumeral deformity secondary to brachial plexus birth palsy J Bone Joint Surg Am 2006 8 3 564 574 10.2106/JBJS.D.02872 16510824

